# Fluoride bioavailability in saliva and plaque

**DOI:** 10.1186/1472-6831-12-3

**Published:** 2012-01-09

**Authors:** Ella A Naumova, Phillip Kuehnl, Philipp Hertenstein, Ljubisa Markovic, Rainer A Jordan, Peter Gaengler, Wolfgang H Arnold

**Affiliations:** 1 Faculty of Health, Department of Dentistry, University of Witten/Herdecke, Alfred Herrhausenstrasse 50, 58448 Witten, Germany

## Abstract

**Background:**

Different fluoride formulations may have different effects on caries prevention. It was the aim of this clinical study to assess the fluoride content, provided by NaF compared to amine fluoride, in saliva and plaque.

**Methods:**

Eight trained volunteers brushed their teeth in the morning for 3 minutes with either NaF or amine fluoride, and saliva and 3-day-plaque-regrowth was collected at 5 time intervals during 6 hours after tooth brushing. The amount of collected saliva and plaque was measured, and the fluoride content was analysed using a fluoride sensitive electrode. All subjects repeated all study cycles 5 times, and 3 cycles per subject underwent statistical analysis using the Wilcoxon-Mann-Whitney test.

**Results:**

Immediately after brushing the fluoride concentration in saliva increased rapidly and dropped to the baseline level after 360 minutes. No difference was found between NaF and amine fluoride. All plaque fluoride levels were elevated after 30 minutes until 120 minutes after tooth brushing, and decreasing after 360 minutes to baseline. According to the highly individual profile of fluoride in saliva and plaque, both levels of bioavailability correlated for the first 30 minutes, and the fluoride content of saliva and plaque was back to baseline after 6 hours.

**Conclusions:**

Fluoride levels in saliva and plaque are interindividually highly variable. However, no significant difference in bioavailability between NaF and amine fluoride, in saliva, or in plaque was found.

## Background

Already two decades ago it has been postulated that site-specific aspects of salivary fluoride clearance may have important implications for the site-specificity of oral diseases [1]. It is now well known that at least three factors are influencing this site-specificity of oral pathobiology: the different local composition and pathogenicity of oral biofilms (local microbiome), the site-specific host response towards bacterial phylotypes as commensals or pathogens (local immunity), and finally, the individual variability of salivary and plaque clearance of fluoride.

Whereas the first two factors are exclusively in the focus of basic research, the kinetics of fluoride in oral fluids are rather well documented. This is the reason why clinical recommendations for the treatment of incipient caries lesions or for the stagnation of lesions can be concluded. Therefore, the bioavailability of fluoride in saliva, and consequently in plaque fluid plays a crucial role in preventing a net mineral deficit in enamel, cementum and dentin due to caries challenge.

Bioavailability of fluoride is dependent upon various factors such as fluoride administration [2-7], fluoride formulation, and salivary secretion rate [8-11]. Fluoride bioavailability in plaque may also be influenced by the compounds of the administered fluoride source. Recently it has been demonstrated, that e. g. sodium lauryl sulphate changes the structure of plaque biofilms which may have an effect on fluoride uptake or release [12]. Several studies have demonstrated, that salivary fluoride concentration increases dramatically after fluoride administration either after tooth brushing or mouth rinsing with fluoridated products, but is back to the baseline level two hours after fluoride administration [4,9,13,14].

Fluoride concentration in saliva is the source for the fluoride delivery to dental plaque. Recently it has been demonstrated that elevated fluoride products like dentifrices with 5000 ppm NaF or amine fluoride [10,15] or oral hygiene tablets directly dissolved in saliva with 4350 ppm NaF enhance remineralization of advanced enamel lesions [16] and result in increased bioavailability of fluoride in saliva [9]. Application of high concentrations of fluoride leads to the formation of a CaF_2 _layer on the enamel surface. It has been reported that this CaF_2 _layer dissolves rapidly and releases bioavailable fluoride [17].

No calcium-fluoride-like deposits were detected in plaque shortly after a NaF mouth rinse [18], and the authors concluded that the inability to form more persistent CaF_2 _deposits may account for the rapid loss of fluoride in plaque after the use of topical fluoride agents. Concerning the plaque clearance of fluoride representing consequently the F^- ^bioavailability over the day and night time rather controversial results have been reported: An experimental ex-vivo study demonstrated a rapid and very substantial uptake of fluoride by plaque after exposure to 1000 ppm NaF immersion [19], whereas from an in-vivo study it was concluded that elevated salivary fluoride concentrations were not reflected in dental plaque, measured 6 h after brushing (1400 ppm fluoride) and rinsing (250 ppm fluoride) [8]. Other data demonstrated after one hour post brushing (1074 ppm fluoride) a rapid fluoride uptake and 12 hours later a clearance back to the placebo levels [20]. More detailed fluoride kinetics data in dental plaque are missing.

As fluoride binding to the plaque reservoirs and the release from the reservoir is rather complex, the source of the fluoride may play an important role. It is well known that different fluoride formulations lead to different salivary fluoride concentrations after tooth brushing [21]. NaF is instantly dissociating in saliva. Sodium monofluorphsphate (NaMFP) requires hydrolysis to release free fluoride ions [11], and amine fluoride may bind to organic constituents in saliva and plaque and releases fluoride slower than the other two. Higher fluoride concentrations may result in the formation of a CaF_2 _layer on the enamel surface which also may serve as fluoride reservoir [17]. The different dissolution properties may lead to different fluoride concentrations in plaque, consequently affecting the caries protective effect of plaque fluoride content.

It was, therefore, the aim of the present investigation to follow up the fluoride bioavailability in whole saliva and in individual plaque samples from baseline immediately after tooth brushing and up to 360 minutes, to compare a NaF formulation dissolved directly in saliva with an amine fluoride dentifrice formulation. The null hypothesis that there is no difference in the fluoride bioavailability after NaF or amine fluoride application was tested.

## Methods

### Subjects

A power analysis with a power of 0.8 at a significance level of p = 0.05 prior to the investigation resulted in a minimum of 6 individuals and three samples per individual to gain reliable data. The data for the power analysis relayed on data obtained in a previous study [9]. Eight healthy subjects participated in this crossover study (7 male and 1 female subject, 24 - 65 years of age). They consented after verbal and written information on the aim and performance of the investigation and also received written instructions and a schedule. Participants were further asked to avoid fluoride-rich food products such as tea, fish and specified mineral water during the period but had no restriction concerning drinking water. All test subjects were residents in the area with ≈ 0.2 ppm fluoride in the drinking water and normally used fluoride containing dentifrices twice daily. The participants had good oral health. Prior to the inclusion into the study salivary flow rate was determined and only normal secretors (0.25 - 1.0 ml/min) were included. The study protocol was approved by the Ethical Committee of the University of Witten/Herdecke, Germany (permission 21/2008).

### Fluoride products

NaF was administered as oral hygiene tablets DENTTABS ^® ^(Innovative Zahnpflegegesellschaft mbH, Berlin, Germany) containing 1450 ppm fluoride per 1.0 g tablet. The tablet had to be chewed before tooth brushing, and the teeth were brushed with a wet tooth brush. Amine fluoride was administered as dentifrice ELMEX ^® ^(Gaba, Lörrach, Germany) containing 1400 ppm fluoride from Olaflur ^®^.

### Study design

Before the experiments all participants received professional dental cleaning. Then they abstained from any oral hygiene, and plaque was grown for three days prior to the experiments. All participants brushed their teeth in the lower jaw in the morning for 3 minutes with either NaF or amine fluoride formulations. Whole saliva and plaque was collected at 5 time intervals for 6 hours. Immediately before brushing (T0) and 3 (T1), 30 (T2), 120 (T3) and 360 (T4) minutes after tooth brushing saliva was collected by spitting into plastic tubes for 3 minutes. Plaque was collected from the upper jaw teeth at the same time intervals with a sterile curette from the approximal sites of molars and premolars strictly from one given site. Therefore, the plaque samples were not pooled. These 5 plaque samples represented the 3-day plaque regrowth on 3 interproximal buccal sites (teeth 14 - 17) and 2 interproximal palatinal sites (teeth 14 - 16).

All subjects repeated every cycle 4 times with both formulations (cross over). Individual cycles with plaque amount less than 1 mg per sample were excluded and consequently repeated. The plaque weight used was between 1 mg and 5.4 mg. The washout period between each cycle was one week. During the washout period the subjects were allowed to perform their individual oral hygiene procedures.

### Fluoride determination

After removal of plaque the weight was determined with a precision balance and the plaque samples were diluted in 500 μl TISAB III (Thermo Electron, Beverly, MA, USA). To compare plaque and saliva values whole saliva samples were weighted, and then centrifuged (B Centrifuge, Beckman Instruments Inc., Germany) for 10 min at 6000 rpm in micro- centrifuge tubes. An aliquot of 1 ml was taken and mixed with 1 ml of a TISAB II buffer solution (Thermo Electron, Beverly, MA, USA). For fluoride ion distribution during the measurement a magnetic stick stirrer (size 2 × 5 mm) was used. The salivary fluoride content was analyzed using a fluoride-sensitive electrode (96-09 Orion, Thermo Electron, Beverly, MA, USA). All measurements were repeated three times and the mean of the measurements was calculated and used for further statistical evaluation.

For the measurement of the fluoride content the following analytical techniques were used: direct calibration and incremental techniques (the method of known addition for low ionic strength samples with a fluoride concentration of less then 0.38 ppm) [22]. Direct calibration was performed in a series of prepared standards of 0.4, 4.0, 40 and 400 ppm fluoride.

### Statistical Methods

The obtained data were processed with the Statistical Package for Social Sciences (SPSS 15.0, Chicago, III., USA). The post-brushing values at the different time intervals were compared with baseline levels using the nonparametric singn test for related variables. As four tests have been applied on the data for the time intervals, the Bonferroni correction was applied and resulted in a p-value of p < 0.0125 for those tests. For comparison of the total amount of fluoride in saliva and plaque after NaF or Amine fluoride administration, curves were plotted for every test person for all time intervals and the area under curve was calculated. These data were then compared with the non parametric Wilcoxon-Mann-Whitney-Test for independent variables. The level of significance for the comparison between NaF and Amine fluoride was 0.05.

## Results

**Figure 1:**
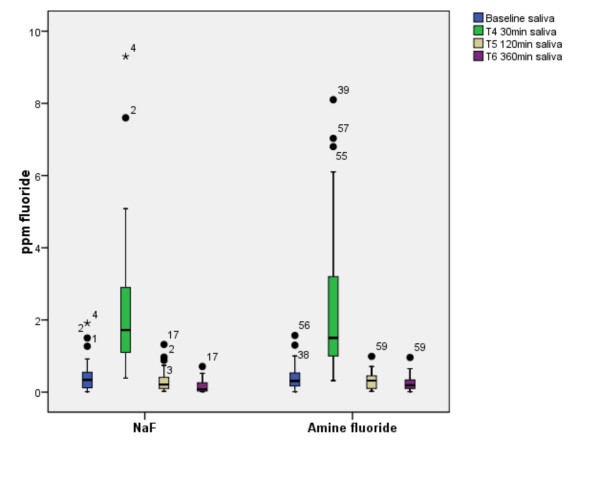
**Fluoride concentration in saliva**. Fluoride concentration in saliva at baseline, 30, 120 and 360 minutes after tooth brushing. The boxplots demonstrate the high interindividual variability demonstrated by the extend of the whiskers and the extremes of the salivary fluoride content.

**Figure 2:**
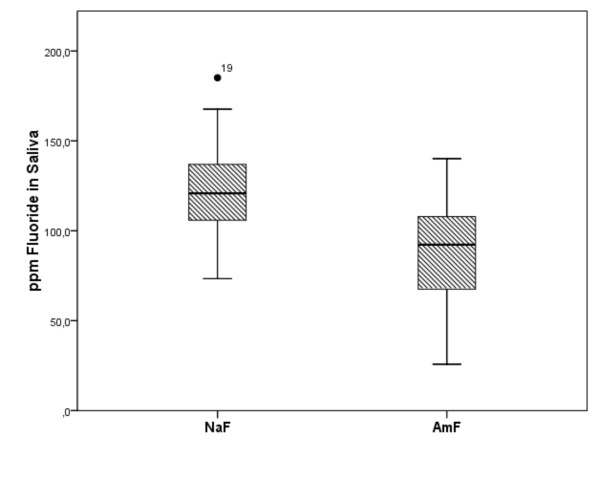
**Total amount of fluoride in saliva**. The boxplot graphic of the total amount of fluoride over the whole measured time period demonstrates a significantly higher salivary fluoride content after NaF administration which can be seen by the higher value of the median in the NaF group.

The baseline fluoride content of saliva ranged in the 8 cycles per subject, in a total of 64 measurements, from 0.02 ppm to 1.93 ppm. The mean fluoride content was 0.41 ppm ± 0.38 ppm for both study arms, and the baseline levels for NaF and amine fluoride were statistically not different (Table 1). The salivary fluoride concentration for the NaF study arm immediately after brushing was higher compared to amine fluoride (p = 0.017). The range of the fluoride content was 100.0 to 264.0 ppm for Na F and 70.0 to 183.0 ppm for amine fluoride. Thirty minutes after brushing the fluoride concentration was still elevated about 10 fold compared to the baseline values, but not significantly (p = 0.73). The range for the NaF arm was 0.4 to 9.3 ppm for NaF and 0.3 to 8.1 ppm for amine fluoride Two hours after brushing the fluoride content in saliva was back to baseline and the rather high interindividual and intraindividual range was demonstrated also for the 6 hours measurements after tooth brushing in both study arms (Figure 1). Comparison of the total salivary fluoride content from baseline until 6 hours after tooth brushing demonstrated a significantly higher fluoride in saliva for NaF (p < 0.001) (Figure 2).

**Table 1:** Salivary fluoride content (in ppm) after tooth brushing with NaF or amine fluoride

	Baseline			3 minutes			30 minutes			120 minutes			360 minutes		
	**Mean**	**STD**	**p**	**Mean**	**STD**	**p**	**Mean**	**STD**	**p**	**Mean**	**STD**	**p**	**Mean**	**STD**	**p**

Amine fluoride	0.39	0.35	p = 0.68	120.7	25.81	p = 0.017	2.3	1.9	p = 0.73	0.31	0.22	p = 0.51	0.24	0.19	p = 0.03
									
NaF	0.44	0.42		171.1	38.8		2.3	1.9		0.30	0.29		0.16	0.18	

The baseline individual plaque fluoride content ranged from 3.9 to 676 ppm, and the mean for all 8 subjects was 147.5 ± 171.1 ppm. All baseline levels of interproximal plaque samples were statistically not different in both study arms (Table 2). Immediately after brushing the fluoride content in plaque did not increase, and the individual range from subject to subject and from cycle to cycle was as high as the baseline data.

**Table 2:** Fluoride content (in ppm) in plaque after tooth brushing with NaF of amine fluoride

	Baseline			3 minutes			30 minutes			120 minutes			360 minutes		
	**Mean**	**STD**	**p**	**Mean**	**STD**	**p**	**Mean**	**STD**	**p**	**Mean**	**STD**	**p**	**Mean**	**STD**	**p**

Amine fluoride	147.5	171.1	p = 0.93	128.5	139.3	p = 0.5	192.6	209.6	p = 0.56	177.7	154.8	p = 0.74	130.4	98.08	p = 0.39
									
NaF	161	217.9		170.4	161.2		220.2	217.9		216.1	438.5		164.3	186.04	

**Figure 3:**
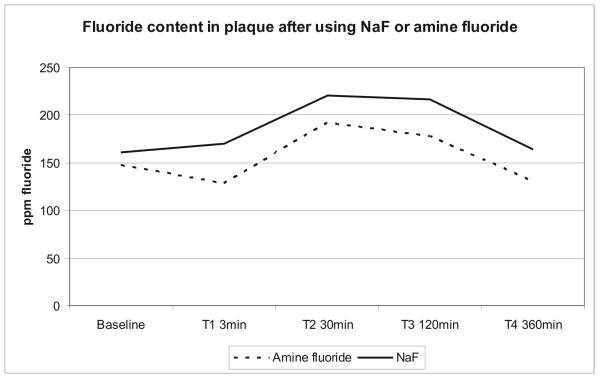
**Fluoride content in plaque**. Fluoride content in plaque after brushing with NaF or amine fluoride. The fluoride content in plaque is increasing 30 and 120 minutes after using NaF or amine fluoride and dropping to baseline level after 360 minutes. The differences are not significant.

**Figure 4:**
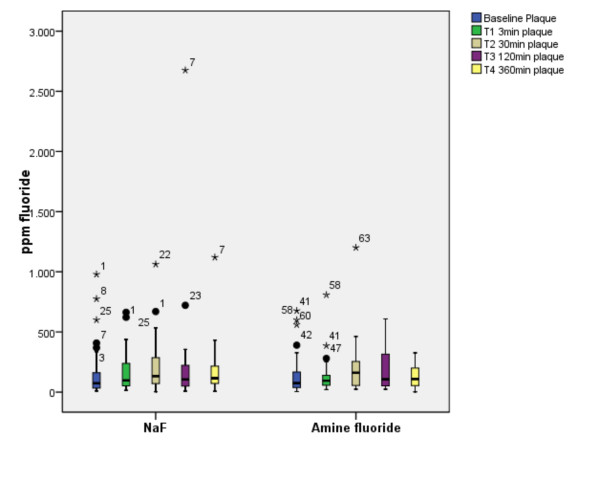
**Variability of fluoride content in plaque**. The boxplot graphic demonstrated the high interinidividual variability of plaque fluoride content after administration of NaF or amine fluoride demonstrated by the extend of the whiskers and the extremes. There is not significant difference between the different time intervals and between NaF and amine fluoride.

The fluoride content in plaque increased 30 minutes after tooth brushing, but the increase was not significant (p = 0.152). The range of the NaF arm was 3 to 1063 ppm, and for the amine fluoride arm was 24.3 to 1201 ppm fluoride. Between 30 minutes and 2 hours the fluoride content within plaque decreased slightly and after 6 hours the fluoride content in plaque was close to the baseline level with no significant differences (Figure 3). Six hours after tooth brushing the mean fluoride content in plaque and the high intraindividual and interindividual range represented the baseline levels with no statistical differences (Figure 4)

## Discussion

Several studies have shown that after fluoride administration, either with dentifrice or mouth rinse, salivary fluoride concentration increases shortly after administration and drops back to the baseline level after 3 to 6 hours [8-11,23]. It is well known that fluoride penetrates into plaque by diffusion [19], and thus becomes a fluoride reservoir which stores fluoride for some time and releases fluoride [18,24]. Plaque fluoride content depends mainly upon the time of exposure to fluoride [20,25,26] and the fluoride formulation [11,21,27,28].

The results of the present study demonstrated a peak increase of salivary fluoride concentration immediately after brushing and lasting for at least 30 min. This is about the time for fluoride diffusion into the plaque biofilm [19], and consequently the plaque fluoride concentration is elevated 30 min after brushing. Both formulations, NaF and amine fluoride, demonstrated the same trend, whereas the fluoride concentration after NaF administration was slightly higher. These results are confirmed by the assessment of penetration of fluoride into natural biofilms. From the literature it is known that the fluoride uptake into plaque is restricted after short term exposure up to 120 sec, whereas exposure for 30 min. demonstrated significantly higher concentrations even in deep plaque layers towards the enamel surface [19]. The plaque fluoride content is of great importance, since dental plaque bacteria are responsible for causing caries. Fluoride is enhancing reminaralization of the enamel surface. The fluoride content in dental plaque may also be dependent upon the fluoride formulation.

All 8 subjects exhibited a rather normal distribution of fluoride concentration at baseline and still 2.5 to 10 fold increase after 30 min. throughout the two study arms there are subjects with a rather constant fluoride bioavailability in plaque (from baseline to increase after 30 min. and back to baseline after 360 min.) on the one hand, and, on the other hand subjects with very variable fluoride concentrations in plaque (as well as at baseline, increase after 30 min. as after 360 min.). This clinical study clearly demonstrated high intraindividual and interindividual as well as site-specific differences in the salivary and plaque fluoride bioavailability. According to the research protocol and considering the limitations of this study, no differences between NaF and amine fluoride were observed for fluoride concentrations in saliva and plaque. Thus the null hypothesis has been confirmed. The high interindividual variability has been demonstrated before [9,29] and may be a reason for the non significant differences in the fluoride content between NaF and amine fluoride.

## Conclusions

There are obviously many factors contributing to the oral fluoride kinetics in an open organ system like the oral cavity. Saliva secretion and content plays a major role for bioavailability of fluoride [9,29]. But there are other factors such as oral hygiene behaviour, brushing time and frequency, fluoride formulation and bioavailability of acting ions, dietary tradition and alimentary fluoride sources and seemingly unknown factors which contribute to the efficiency of bio available fluoride. To enlighten the complex interplay between saliva, plaque and fluoride bioavailability further in vivo and in vitro studies experimental have to be carried out.

## Competing interests

The authors declare that they have no competing interests.

## Authors' contributions

EAN did the planning of the project and supervision; PK was responsible for the plaque collection and fluoride measurement in the plaque samples; PH was responsible for saliva collection and fluoride measurement in saliva: LM provided the patients for plaque and saliva collection; RAJ also provided patients for plaque and saliva collection; PG was advisor of the project; WHA wrote manuscript and did the statistical calculation. All authors read and approved the final manuscript.

## Pre-publication history

The pre-publication history for this paper can be accessed here:

http://www.biomedcentral.com/1472-6831/12/3/prepub
